# A familial case of alveolar capillary dysplasia with misalignment of the pulmonary veins: the clinicopathological features and unusual glomeruloid endothelial proliferation

**DOI:** 10.1186/s13000-020-00972-6

**Published:** 2020-05-09

**Authors:** Akiko Kitano, Masato Nakaguro, Seiichi Tomotaki, Shintaro Hanaoka, Masahiko Kawai, Akiko Saito, Masahiro Hayakawa, Yoshiyuki Takahashi, Hidenori Kawasaki, Takahiro Yamada, Masahiko Ikeda, Tetsuo Onda, Kazutoshi Cho, Hironori Haga, Atsuko Nakazawa, Sachiko Minamiguchi

**Affiliations:** 1 Department of Diagnostic Pathology, Kyoto University Hospital, 54 Kawahara-cho, Sakyo-ku, Kyoto, 606-8507 Japan; 2 Department of Pathology and Laboratory Medicine, Nagoya University Hospital, Nagoya, Japan; 3 Department of Pediatrics, Graduate School of Medicine, Kyoto University, Kyoto, Japan; 4 Division of Neonatology, Center for Maternal-Neonatal Care, Nagoya University Hospital, Nagoya, Japan; 5 Department of Pediatrics, Nagoya University Graduate School of Medicine, Nagoya, Japan; 6 Clinical Genetics Unit, Kyoto University Hospital, Kyoto, Japan; 7 Maternity and Perinatal Care Center, Hokkaido University Hospital, Sapporo, Japan; 8 Department of Clinical Research, Saitama Children’s Medical Center, Saitama, Japan

**Keywords:** Alveolar capillary dysplasia, Case report, Familial, *FOXF1*, Glomeruloid endothelial proliferation, Misalignment of pulmonary vein

## Abstract

**Background:**

Alveolar capillary dysplasia with misalignment of pulmonary veins (ACD/MPV) is a rare disorder of pulmonary vascular abnormality with persistent pulmonary hypertension of the newborn. The symptom usually presents within hours after birth, leading to an early demise. Heterozygous de novo point mutations and genomic deletions of the *FOXF1* (forkhead box F1) gene or its upstream enhancer have been identified in most patients with ACD/MPV. Most cases of ACD/MPV are sporadic; however, familial cases are also reported in 10% of patients.

**Case presentation:**

We herein report a case of familial ACD/MPV that showed unusual glomeruloid proliferation of endothelial cells. In this family, three of the four siblings died within two to 3 days after birth because of persistent pulmonary hypertension and respiratory failure. Only the second child remains alive and healthy. An autopsy was performed for the third and fourth children, resulting in a diagnosis of ACD/MPV based on the characteristic features, including misalignment of smaller pulmonary veins and lymphangiectasis. In both of these children, glomeruloid endothelial proliferation of vessels was noted in the interlobular septa. The vessels were immunohistochemically positive for D2–40, CD31, Factor VIII, and ERG, suggestive of differentiation for both lymphatic and blood vessels.

**Conclusions:**

Unusual glomeruloid endothelial proliferation was observed in a familial ACD/MPV case. This histologic feature has not been described previously in ACD/MPV or any other pulmonary disease. Although the histogenesis of this histologic feature is unclear, this finding may suggest that ACD/MPV is a compound vascular and lymphovascular system disorder that exhibits various histologic features.

## Background

Alveolar capillary dysplasia with misalignment of pulmonary veins (ACD/MPV) is a rare, fatal developmental disorder of the pulmonary blood vessels. It presents with persistent pulmonary hypertension of the newborn (PPHN) and severe hypoxemia, leading to progressive respiratory failure and an early demise [1, 2]. PPHN is refractory to all standard medical therapies including extracorporeal membrane oxygenation [3]. The primary characteristic histologic feature of ACD/MPV is the anomalous location (misalignment) of smaller pulmonary veins (MPV), which run adjacent to the bronchioles and pulmonary arteries within a common adventitial sheath. In addition, a decreased number of pulmonary capillaries, thickened alveolar septa, medial hypertrophy of small pulmonary arteries, and lymphangiectasis are reported histologic features of ACD/MPV in the literature [4–6]. Roughly 50–80% of patients also show extrapulmonary anomalies in the gastrointestinal, cardiovascular and urogenital systems [1, 7]. Genetically, heterozygous de novo point mutations and *FOXF1* (forkhead box F1) genomic or its upstream deletion have been reported [8–12]. While many of the reported cases are sporadic, approximately 10% of ACD/MPV cases have a familial association [11–14].

We herein report the clinicopathologic features of a case of familial ACD/MPV exhibiting unusual glomeruloid endothelial proliferation and discuss the significance of these findings.

## Case presentation

**Fig. 1:**
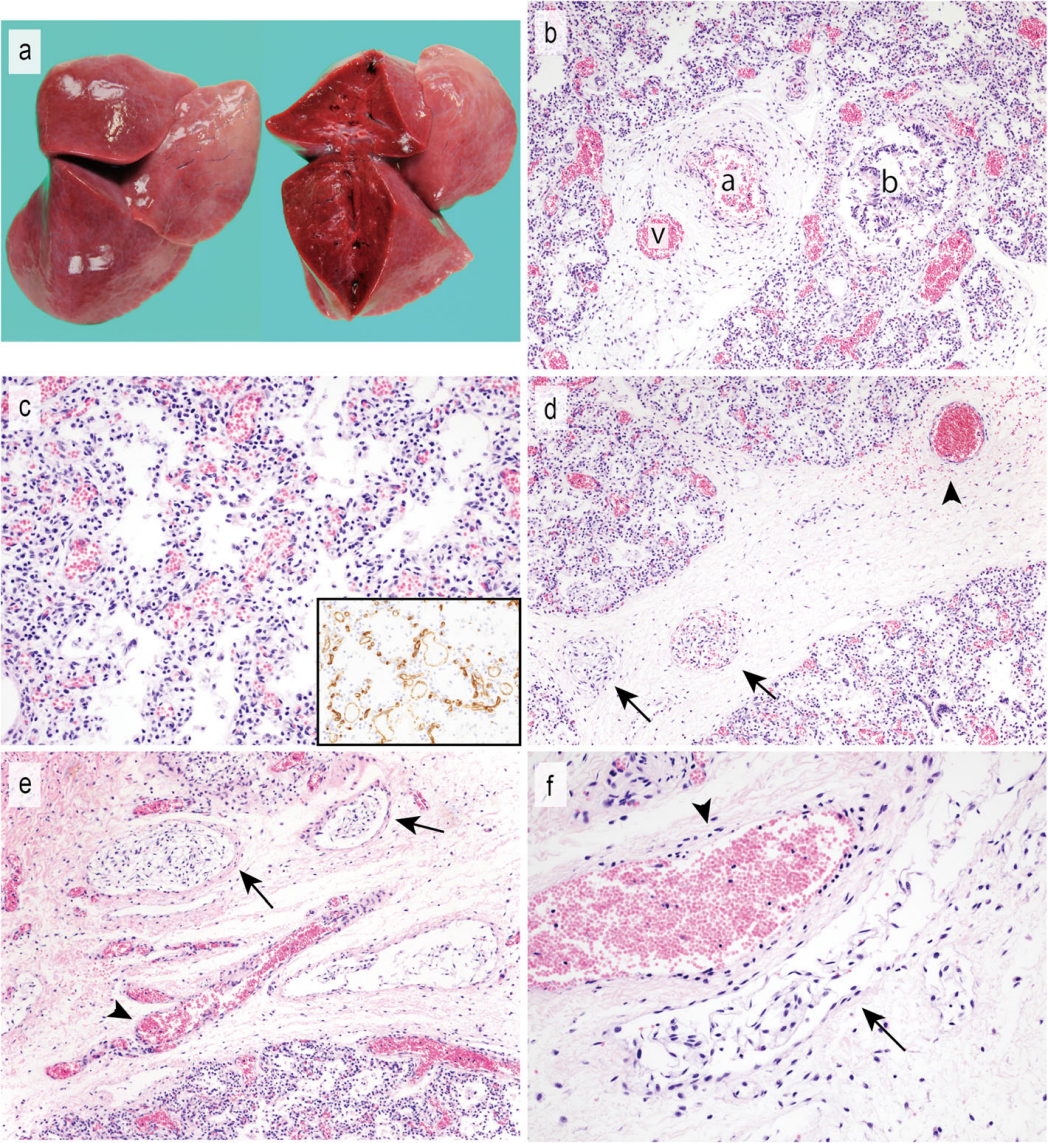
Histologic and immunohistochemical features of the third child. **a** A macroscopic image (left) and the cut surface (right) of the right lung before formalin fixation. Congestion and loss of elasticity were noted. **b** A misaligned pulmonary vein (v) is adjacent to a pulmonary artery within the same adventitial sheath (a). A bronchiole (b) abuts the vein and artery. **c** Thickening of the alveolar wall. Immunohistochemical staining for CD34 highlights the dilation of the capillary of the alveolar septa (inset). In this case, a decrease in the number of alveolar capillaries is not remarkable. **d**-**f**) Low-power (**d**, **e**) and high-power (**f**) views of the interlobular septum. Vessels with glomeruloid endothelial proliferation (arrow) contrast with blood vessels (arrowhead). The vessels are dilated and contain no or few red blood cells

**Fig. 2:**
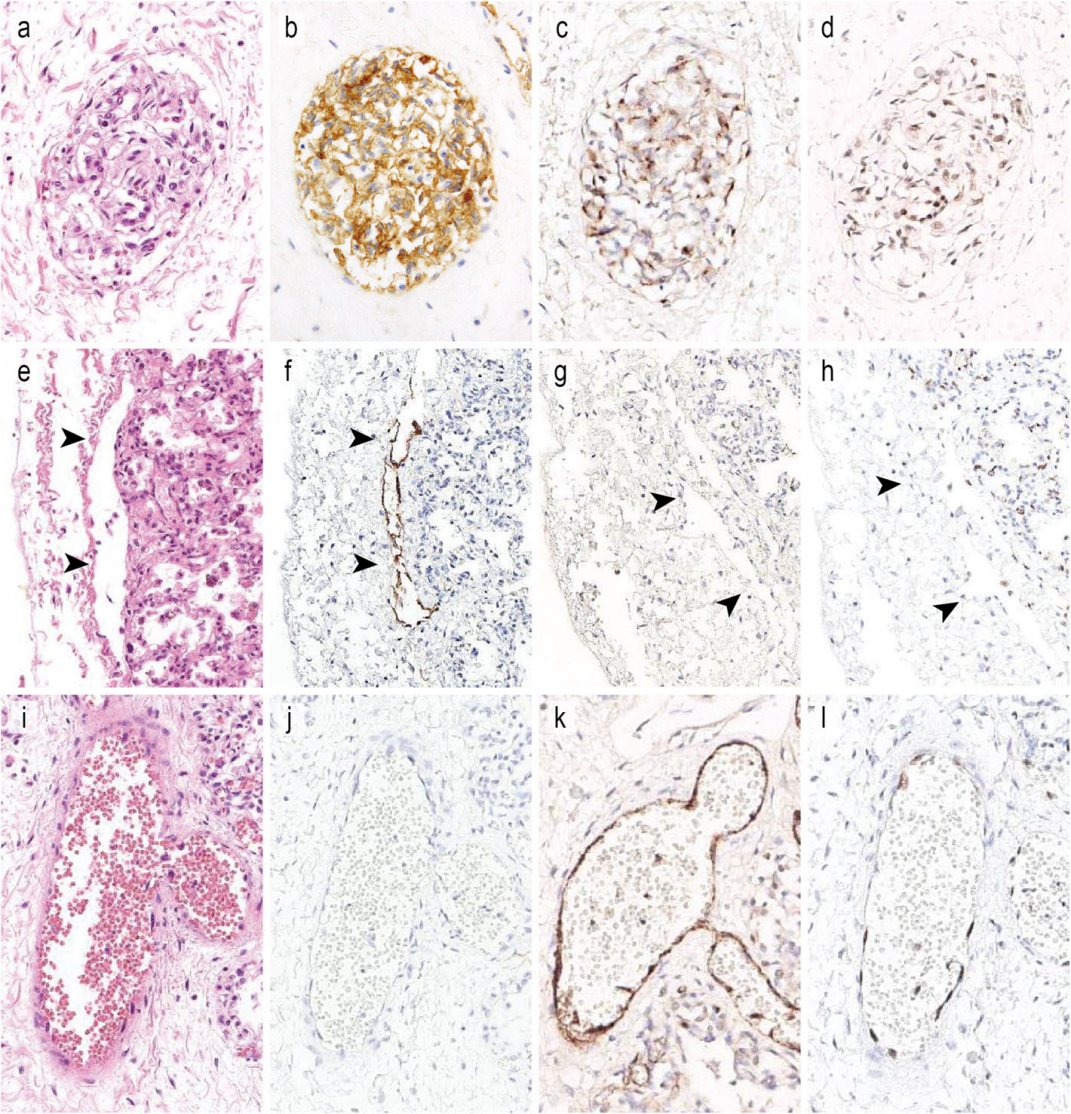
Immunohistochemical results of glomeruloid endothelial proliferation (**a-d**), pleural lymphatic vessels (**e-h**), and blood vessels in interlobular septum (**i-l**) for D2–40 (**b**, **f**, **j**), Factor VIII (**c, g, k**), and ERG (**d, h, l**). Glomeruloid endothelial proliferation expressed both lymphatic marker (D2–40) and blood vessel markers (Factor VIII and ERG). (**a-d**, **i-l**: the third child, **e-h**: another neonatal autopsy case that died from other causes)

**Fig. 3:**
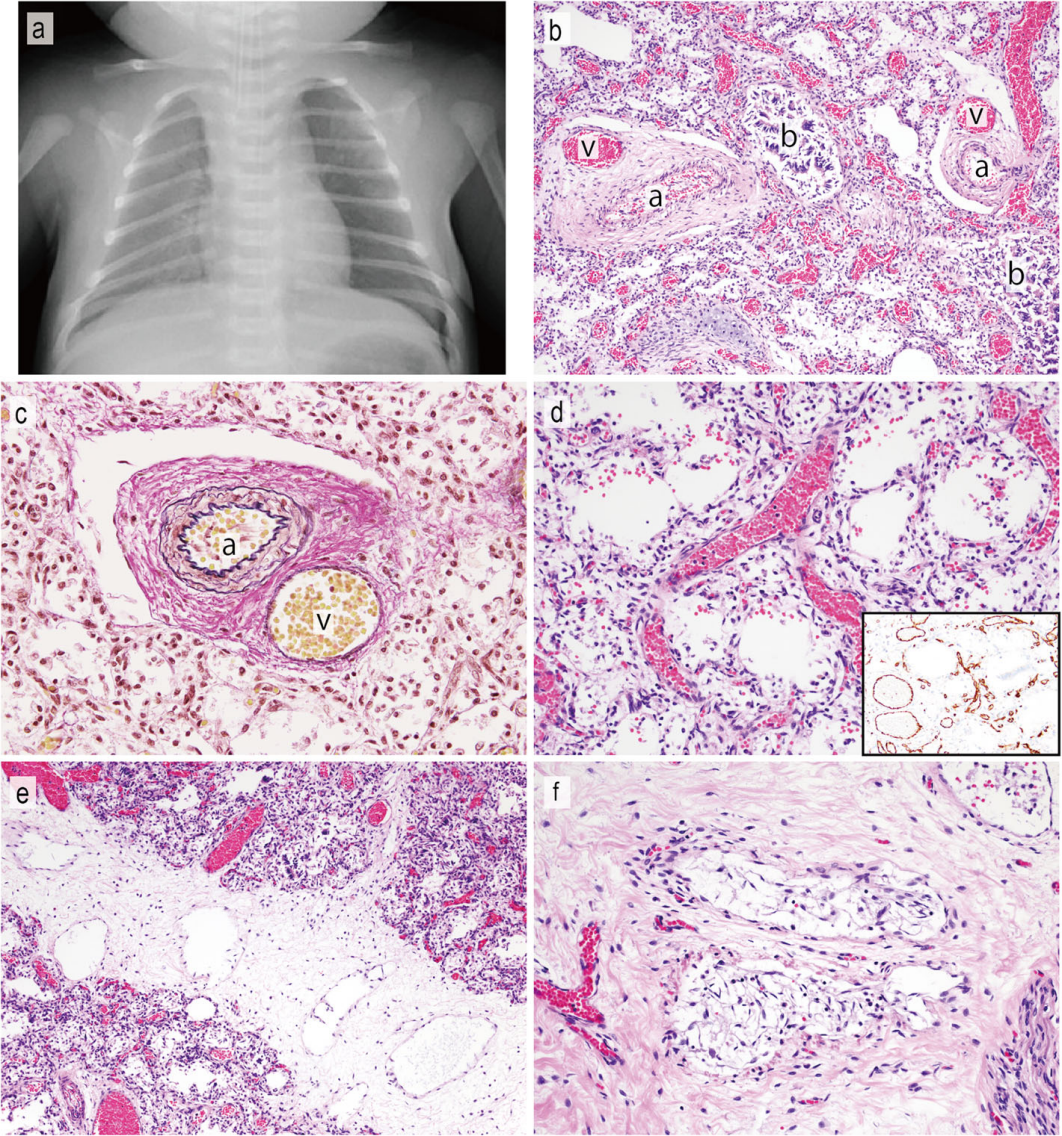
Radiographic and histopathological findings of the fourth child. **a** Chest X-ray reveals a diffuse decrease in translucency in the bilateral lung fields. **b** Misaligned pulmonary veins (v) run alongside the small pulmonary arteries (a) within a common adventitial sheath and a bronchiole (b). **c** Elastica van Gieson staining highlights the malposition of a pulmonary vein and artery. **d** Thickening of the alveolar wall and dilation of vessels. Immunohistochemical staining for CD31 shows a decreased number of pulmonary capillaries located away from the alveolar epithelium (inset). **e** Most of the lymphatic vessels in the interlobular septa are dilated without endothelial proliferation, and glomeruloid endothelial proliferation was limited to a small area (**f**)

The radiographic and macro- and microscopic features of the third and fourth children are shown in Figs. 1, 2 and 3.

### Clinical history

In this family, three of four siblings born to nonconsanguineous parents died within a few days after birth because of PPHN and respiratory failure. An autopsy was performed for the third and fourth children, and the clinical courses of these cases are described below.

The first child was male and born 5 years prior to the fourth child’s birth by Caesarean section due to arrested labor because of cephalopelvic disproportion at 40 weeks’ gestation. The mother’s age was 30 years old. There were no problems during the pregnancy. The birth weight was 3592 g, and the Apgar scores were 8/9. However, meconium staining of the amniotic fluid was observed at birth, and he was diagnosed with meconium aspiration syndrome. Immediately after birth, he developed hypoxemia, respiratory distress, pneumothorax and severe PPHN. He was treated with mechanical ventilation, supplemental oxygen, chest drainage, pulmonary vasodilators and vasopressor support. However, despite all efforts, he died at 74 h after birth. An autopsy was not performed for this child.

The second child was male and born 4 years prior to the fourth child’s birth by planned Caesarean section at 38 weeks’ gestation. The birth weight was 3152 g and he is alive and healthy without any respiratory problems.

The third child was male and born 2 years prior to the fourth child’s birth by planned Caesarean section at 38 weeks’ gestation. There were no problems during the pregnancy. The birth weight was 3696 g, and the Apgar scores were 9/10. At about 3 h after birth, he developed cyanosis and hypoxemia, showing a percutaneous oxygen saturation (SpO_2_) of 60%. His respiratory distress was unresponsive to mechanical ventilation, supplemental oxygen or vasopressor support. He died at 37 h after birth. An autopsy was performed for this child due to suspicion of congenital interstitial lung diseases.

The fourth child was female and born by planned Caesarean section at 38 weeks’ gestation. The mother was 36 years old and had gestational diabetes. The birth weight was 3300 g, and the Apgar scores were 8/8. The infant had postaxial polydactyly on her left foot but no other external malformations. She developed tachypnea at 4 h after birth, and standard therapies of mechanical ventilation, inhaled nitric oxide and vasopressor support were started. Chest X-ray revealed decreased translucency in the lung field (Fig. 3a). Hypoxemia showed temporary improvement, with the SpO_2_ increasing to 100%, but she still presented with PPHN. Despite these therapies, she died of respiratory failure at 40 h after birth, and an autopsy was performed.

Although a familial association was strongly suggested in this case, no similar neonatal deaths had not been observed in other relatives.

### Pathologic findings

An autopsy of the third child was performed 54 h after his death. The autopsy revealed a male infant measuring 52 cm in height and weighing 5130 g. No external or internal malformations were detected. Bilateral pleural effusion (100 ml each) and ascites (350 ml) were found, and the left and right lungs weighed 41.6 g and 49.5 g, respectively. The bilateral lungs were congested and had lost their elasticity (Fig. 1a). Histologically, the diagnosis of ACD/MPV was confirmed by the presence of MPV (Fig. 1b). Other characteristic histologic features of ACD/MPV, such as thickening of alveolar wall (Fig. 1c), hypertrophy of small pulmonary arteries, and lymphangiectasis were also observed. In the interlobular septa, glomeruloid endothelial proliferation was observed in dilated vessels (Figs. 1d-f). The endothelial cells showed no cellular atypia. Although the vessels were more morphologically similar to lymphatic vessels than blood vessels (vessel with thin walls and no or few red blood cells inside), immunohistochemically, the endothelial cells were positive for D2–40, CD31, Factor VIII, and ERG (Fig. 2). These immunohistochemical results suggested that the vessel had dual differentiation for lymphatic and blood vessels. Blood vessels in the interlobular septa were not remarkable in number, size or structure (Figs. 1d-f). Except for liver congestion, no remarkable findings in other organs were detected.

An autopsy of the fourth child was performed at 82 h after her death. The autopsy revealed a female infant measuring 47 cm in height and weighing 3000 g. An external examination showed an extra toe on her left foot. Pleural effusion and ascites were not found, and no internal malformation was identified macroscopically. The left and right lungs weighed 28.0 g and 33.5 g, respectively, which was within the reference value. In the cut surfaces of the lung, no noticeable findings were observed macroscopically except for small blebs under the pleura of the left upper lung. Histologically, MPV (Figs. 3b, c), a decrease in the number of pulmonary capillaries, thickened alveolar septa (Fig. 3d), lymphangiectasis (Fig. 3e), and medial hypertrophy of small pulmonary arteries were observed in all lobes of the lung. The glomeruloid endothelial proliferation in the interlobular septa that was observed in the third child was present in a limited area (Fig. 3f). Immunohistochemical studies revealed the same results. There were no abnormalities in other organs except for mild liver and splenic congestion. The antibodies used in this study were CD31 (dilution at 1:1200; Dako, Carpinteria, CA), D2–40 (dilution at 1:500; Dako, Carpinteria, CA), Factor VIII (pre-diluted; Nichirei Biosciences Inc., Tokyo, Japan), and ERG (dilution at 1:100; Abcam, Cambridge, UK).

Tissue samples from the third and fourth children were genetically analyzed. In both cases, a deletion of roughly 450 kbps located about 2840 kbps upstream from exon 1 of the *FOXF1* gene was revealed by multiplex ligation-dependent probe amplification (MLPA) assay.

## Discussion and conclusions

ACD/MPV is a rare disorder of pulmonary vascular development, and over 200 cases have been reported in the literature [2]. A study reported that the estimated incidence of ACD/MPV was approximately 1/100,000 [2]. However, the true incidence is unclear, as histological examinations by an autopsy or biopsy are necessary in order to confirm the diagnosis. Many patients develop respiratory distress and pulmonary hypertension within the first 24 h after birth and die within the first month of life. However, there are several case reports of patients with relatively mild ACD/MPV who present with symptoms after 24 h of life or survive beyond the neonatal period [1, 2, 15, 16]. In these milder ACD/MPV patients, the histologic features are reported to be focal or patchy [15, 16].

Most ACD/MPV cases are sporadic, but about 10% involved siblings and a familial association has been suggested [1, 12–14]. In 2009, Stankiewicz et al. suggested an association between ACD/MPV and mutations of the *FOX* gene cluster on 16q24.1 [8]. Since then, a number of pathogenic variants containing copy number variations, point mutations and a complex rearrangement have been detected, all involving the *FOXF1* gene or its regulatory region [2, 9–12]. While paternal imprinting was suggested in early reports [8, 11], variable clinical presentations in a single family with the same *FOXF1* mutation have suggested somatic mosaicism and complex gene regulation, including unorthodox imprinting of the gene locus [12]. In the present case, we performed genetic testing for the third and fourth children and found the identical deletion of the upstream enhancer region of the *FOXF1* gene. This deletion was previously reported in ACD/MPV patients and included the fetal lung-expressed long noncoding RNA gene *LINC01081*, which positively regulates the FOXF1 expression [17].

ACD/MPV is histopathologically characterized and defined by the presence of MPV [2]. Other histologic features include a decrease in the number of pulmonary capillaries, thickening of alveolar septa, lymphangiectasis, and medial hypertrophy of small pulmonary arteries and muscularization. In the present case, we found these typical histologic features in both children. In addition, glomeruloid endothelial proliferation was observed in the vessels in the interlobular septa (Figs. 1d-f, Fig. 3f). The immunohistochemical results were difficult to interpret. While the pleural lymphatic vessels in the present cases and two other neonatal autopsy cases that died of other causes were immunoreactive for D2–40 and CD31 and negative for Factor VIII and ERG (Figs. 2e-h), the proliferated endothelium was positive for all of these markers (Figs. 2a-d). These results suggested that the vessels could not be simply classified as lymphatic or blood vessels, instead showing differentiation for both lymphatic and blood vessels. These double (lymphatic and blood vessel markers)-positive vessels were reported in a previous study of gastric adenocarcinoma [18]. Although the possibility of immature vasculature was suggested, the origin and significance has not still been elucidated [18]. The vessels of the present cases had thinner walls and no or fewer red blood cells inside than typical blood vessels (Figs. 1d-f, Fig. 3f). The morphology thus appears to be more similar to lymphatic vessels than blood vessels. The previous report of double-positive vessels also found them to be morphologically similar to lymphatic vessels [18]. This unusual glomeruloid endothelial proliferation has not been reported previously in ACD or, to our knowledge, any other pulmonary disorders or neonatal lung.

We compared the distribution and number of lymphatic vessels with the aforementioned two neonate autopsy cases that died from other causes. The number and morphology of pleural lymphatic vessel were not markedly different between the present cases and the non-ADC/MPV autopsy cases. However, in the present cases, the interlobular and intralobular lymphatic vessels, particularly the bronchovascular lymphatic vessels, were dilated and more prominent on D2–40 immunohistochemical staining than non-ACD/MPV cases. This was, although not quantified, caused by the dilation and increase in tortuosity of the lymphatic vessels.

As the histogenesis of the glomeruloid endothelial proliferation, both the possibility of reactive endothelial hyperplasia and primary change caused by ACD/MPV were considered. Given the identical genetic deletion in both children, it was suggested that the lesion was primarily caused by *FOXF1* alteration and that ACD/MPV is a compound vascular and lymphovascular system disorder exhibiting divergent histologic features.

Extrapulmonary congenital anomalies, including cardiovascular, gastrointestinal, and genitourinary systems, have been reported in both sporadic and familial ACD/MPV cases [11]; however, in the present cases, no symptomatic congenital anomalies were detected except for an extra toe in the fourth child.

At present, the definitive diagnosis of ACD/MPV is made by a histologic examination of the lungs, typically at a postmortem autopsy [5]. However, the diagnosis based on a lung biopsy has also been reported recently [16, 19, 20]. In order to confirm the presence of MPV, it is necessary to collect specimens, including blood vessels, by an open lung biopsy. In our case, MPV were distributed diffusely in all lobes, even in the peripheral area (3 mm from the pleura) and an open biopsy could have made a definitive diagnosis if the specimen had been larger than 3 mm in size. However, the clinical course was urgent and a biopsy could not be performed.

In conclusion, we reported a familial case of ACD/MPV with unusual glomeruloid endothelial proliferation. Careful histologic evaluation on lymphatic vessels in future cases and further basic studies are warranted to understand the pathogenesis of ACD/MPD.

## Data Availability

All data generated or analyzed during this study are included in this published article.

## Abbreviations

ACD: Alveolar capillary dysplasia
FOXF1: Forkhead box F1
MPV: Misalignment of pulmonary veins
PPHN: Persistent pulmonary hypertension of the newborn
SpO_2_: Percutaneous oxygen saturation
